# Single-use versus reusable endoscopes in gastroenterology: Systematic review of full and partial economic evaluations

**DOI:** 10.1055/a-2645-1463

**Published:** 2025-07-29

**Authors:** Mandana Zanganeh, Yufei Jiang, Norman Waugh, Anna Brown, Yen-Fu Chen, Ramesh P. Arasaradnam, Lazaros Andronis, on behalf of the "SUMU Endo" project group

**Affiliations:** 1 2707 Centre for Health Economics at Warwick, Warwick Medical School, University of Warwick, Coventry, United Kingdom of Great Britain and Northern Ireland; 2 1019 Institute of Applied Health Sciences, University of Aberdeen, Aberdeen, United Kingdom of Great Britain and Northern Ireland; 3 2707 Warwick Evidence, Warwick Medical School, University of Warwick, Coventry, United Kingdom of Great Britain and Northern Ireland; 4 1724 Birmingham Centre for Evidence and Implementation Science, School of Social Policy and Society, University of Birmingham, Birmingham, United Kingdom of Great Britain and Northern Ireland; 5 2708 Warwick Medical School, University of Warwick, University Hospitals Coventry and Warwickshire NHS Trust, Coventry, United Kingdom of Great Britain and Northern Ireland

**Keywords:** Endoscopy Upper GI Tract, Endoscopy Lower GI Tract, Pancreatobiliary (ERCP/PTCD), Statistics

## Abstract

**Background and study aims:**

Future decision making on health care will need to consider broader environmental and sustainability issues. One example is adoption of single-use endoscopes instead of reusable endoscopes in gastroenterology, largely due to their perceived benefit of reducing cross-infection. Besides considerations related to technical performance, there are differences not only in the cost to healthcare but also in the impact they have on the environment. The primary aim of this systematic review was to synthesize evidence on the costs and consequences of using single-use gastrointestinal endoscopes vs. reusable ones adopting various reprocessing methods. The secondary aim was to review and discuss the way in which environmental impact is costed and reported by the studies included in this review.

**Methods:**

We searched multiple databases and the internet to September 2024. We included and quality-assessed partial and full economic evaluations according to predetermined criteria.

**Results:**

Seven studies (2 cost analyses and 5 cost-utility analyses [CUA]) were included. All focused on duodenoscopes for endoscopic retrograde cholangiopancreatography. Five studies compared single-use with reusable duodenoscopes whereas two studies compared different reprocessing methods for reusable duodenoscopes. The most common outcomes were infection risk (n = 6) and quality-adjusted life years (n = 5). Environmental impact was considered in only two studies. All studies stated that the per-procedure cost was higher using single-use endoscopes but three CUAs indicated that single-use endoscopes were more cost-effective. Several studies used doubtful assumptions, reducing their credibility.

**Conclusions:**

Future economic evaluations of single-use vs. reusable endoscopes require more robust comparative evidence and should include costs and consequences beyond health, especially environmental impact.

## Introduction

Every year, over 22 million endoscopies are undertaken in the United States 1 and approximately 1.5 million in the United Kingdom (UK) 2 . In the UK National Health Service (NHS), most endoscopes are reusable and are used at least hundreds of times after cleaning and disinfection. A recent French study reported usage for an average of 1,280 procedures over the 6-year life span of a reusable gastroscope 3 .

Decontamination processes for reusable devices are essential to prevent cross-infection but require considerable resources and chemicals and, consequently, add to the carbon footprint of endoscopy practice. The NHS aims to deliver a net zero carbon emissions target by 2040 4 . To achieve that, some practices in health care may need to change.

Disposable single-use endoscopes are being marketed on the grounds they reduce or eliminate risk of infection. Their technical performances are comparable to reusable endoscopes 5 6 . However, if we switch to disposable endoscopes, the cost to health care and to the environment may be much greater 7 due to continuous replacement and waste generation. The primary aim of this systematic review is to synthesize evidence on the costs and consequences of using single-use gastrointestinal endoscopes compared with reusable ones in patients undergoing gastrointestinal endoscopy. The secondary aim is to review and discuss the way in which environmental impact is costed and reported by the studies included in this review. To our knowledge, this is the first systematic review of published economic evaluations of single-use vs. reusable gastrointestinal endoscopes.

## Methods

This systematic review follows the reporting guidelines of Preferred Reporting Items for Systematic Reviews and Meta-Analyses (PRISMA) 8 . The protocol is registered with the international prospective register of systematic reviews (PROSPERO) database (reference number CRD42024465642). The study’s Advisory Group included gastroenterologists and health technology assessment experts.

### Search strategy

We searched MEDLINE, Embase, Web of Science, Cochrane Database of Systematic Reviews and CENTRAL, HTA databases (CRD and INAHTA), NHS EED, EconPapers (RePec) and Cost-effectiveness analysis (CEA) Registry up to September 25, 2024, as well as Google Scholar, Google and the NICE, CADTH, and ICER websites. An information specialist (AB) developed search strategies following guidance in the Cochrane Handbook 9 . Searches combined controlled vocabulary terms (e.g. MeSH and EMTREE) with free text keywords and included concepts related to gastrointestinal endoscopy, single-use/disposable, multiple-use/reusable and environmental impact. No language restrictions, date limits, or study type filters were applied. The search strategy was initially developed for searches in MEDLINE (via Ovid) ( **Supplementary Table 1** ) and adapted for other databases. Reference lists of identified studies were checked. Literature search results were managed using EndNote 21 software.

#### Eligibility criteria

Studies were included (or excluded) based on the following criteria:

Format: only full-text articles.

Year: studies published from January 1, 2000.

Language: only studies in English.

Publication status: peer-reviewed journal articles and grey literature. Non-empirical articles (e.g., narrative reviews) were excluded.

Populations: patients undergoing gastrointestinal endoscopy; health care staff involved in the operation, cleaning, reprocessing and disposal of endoscopes.

Intervention: gastrointestinal endoscopy using single-use gastroscopes, sigmoidoscopes, duodenoscopes, colonoscopes. Cholangioscopy was excluded.

Comparator: reusable gastrointestinal endoscopes.

Outcomes: any measure of health (e.g., infections to patients/staff, adverse events [AEs]), patient reported (quality-adjusted life years (QALYs)) and environmental (e.g., carbon footprint); resource use/cost associated with the use of gastrointestinal endoscope (e.g., purchase, reprocessing, repair, treating infection, disposal of endoscopes and their environmental impact).

Study type: full economic evaluations (which consider both costs and consequences as defined by Drummond et al. 10 ) and partial economic evaluations (i.e. cost studies, budget impact analyses).

Environmental effects: studies with or without considerations given to environmental effects. Studies solely focusing on environmental effects were excluded.

Country of origin: any country.

### Study selection procedure

The main researcher (MZ) and NW screened titles and abstracts of identified publications against the selection criteria. If in doubt, articles were checked based on the full text. Full-text papers were reviewed by two researchers (MZ and LA) and a final decision was made with respect to the inclusion/exclusion criteria. Any disagreements over eligibility of specific studies were discussed. If disagreement persisted, input was sought from an additional reviewer (NW).

### Data extraction

Characteristics and findings from the included studies were extracted by one researcher (YJ), using data extraction forms designed for this review with independent checking by another researcher (MZ). Any discrepancies were resolved by discussion or by consensus with an additional reviewer (LA). Extracted information included: 1) details of study (authors, publication year, country, data source, aims/purpose, study population, intervention/s and comparator/s); and 2) a detailed account of the economic evaluation methods and results (type of economic evaluation, model type, study perspective, time horizon, price year/currency, discount rate, outcome measures, resource use/costs, base-case results, sensitivity analyses, generalizability, source of funding, and declared conflicts of interest).

### Quality assessment of included studies

The Consensus on Health Economic Criteria (CHEC) checklist 11 was used by one researcher (YJ) to quality assess included studies with independent checking by a second reviewer (MZ). Any discrepancies were resolved by discussion with an additional reviewer (LA).

The CHEC and other health economic checklists do not assess quality of clinical effectiveness studies from which inputs are obtained. Therefore, we checked key clinical assumptions used in cost-utility modelling. These were the proportion of exogenous and endogenous infections after gastrointestinal endoscopy, the proportion of gastrointestinal endoscopes that are contaminated despite standard disinfection processes, and the proportions of patients who develop clinical infection or colonization after endoscopic procedures using a contaminated endoscope.

## Results

**Fig. 1:**
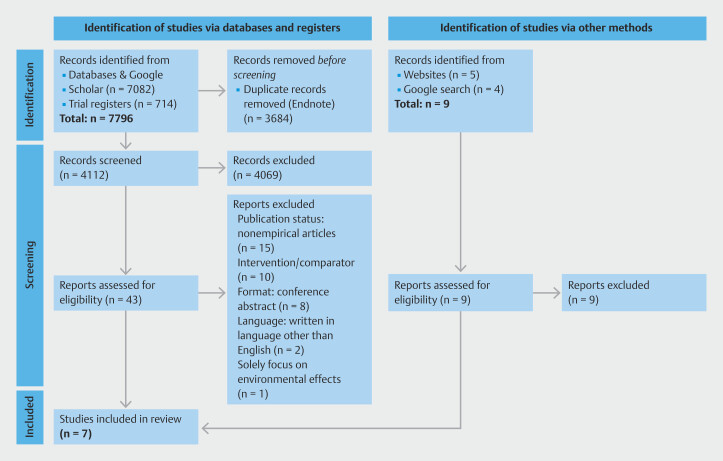
Preferred Reporting Items for Systematic Reviews and Meta-Analyses (PRISMA) flowchart showing the study.

Fig. 1 shows that 7,796 records were identified by the literature search, 4,112 were screened after removal of duplicates, 4,069 were excluded based on titles and abstracts, leaving 43 papers considered potentially relevant for full-text assessment, with a further 36 papers excluded (the most common reasons being non-eligible publication status and non-eligible intervention/comparator [ **Supplementary Table 2** ]), leaving the final set of seven papers 12 13 14 15 16 17 18 .

### Details of study context

Study contexts are presented in Table 1 . Six studies were published between 2020 and 2023 13 14 15 16 17 18 . Four were from the United States 12 13 14 15 , and three from Europe 16 17 18 . In all studies, patients underwent endoscopic retrograde cholangiopancreatography (ERCP) 12 13 14 15 16 17 18 . Five studies 13 15 16 17 18 directly compared single-use duodenoscopes with reusable duodenoscopes whereas two studies 12 14 focused on comparisons between reusable duodenoscopes adopting different reprocessing methods. Four studies specified manufactures (single-use scopes: Boston Scientific 15 17 , Ambu 17 18 ; and reusable scopes: Olympus 16 , Pentax Medical 16 ). Reprocessing methods included high-level disinfection (HLD), double HLD, culture and hold, and ethylene oxide (EtO) sterilization.

**Table:** **Table 1** Summary of general characteristics of the studies.

Authors/ year	Country	Data source	Aims/purpose	Study population	Intervention	Comparator
Almario et al, 2015 12	USA	Hypothetical cohort hospitalized and published studies	To measure cost-effectiveness of four competing strategies for CRE risk management	A hypothetical cohort of patients hospitalized for symptomatic common bile duct stones and underwent ERCP	Four competing strategies (see comparator)	1. ERCP followed by US FDA-recommended reprocessing; 2. ERCP followed by “endoscope culture and hold”; 3. ERCP followed by EtO sterilization of the endoscope; and 4. stop ERCP in lieu of LC with CBDE
Barakat et al, 2022 13	USA	Medical center and published studies	To assess cost of 6 approaches to minimize infection, taking into account duodenoscope-transmitted infection cost	Patients undergoing ERCP in tertiary care academic medical center and sterile processing division. Mean age of patients 60 years (range, 18–99)	PD duodenoscope and FD duodenoscope	Reusable duodenoscope with single HLD, double HLD, EtO sterilization, and culture and hold
Bomman et al, 2021 14	USA	Multicenter study (includes two centers with high ERCP volumes)	To estimate economic impacts of 3 commonly used enhanced-SRT compared to single HLD for performing ERCP	Patients undergoing ERCP in two institutions (Virginia Mason Medical Centre, Seattle and University of California Los Angeles, CA adopted enhanced-SRT)	Three used enhanced-SRT (Double HLD, EtO gas sterilization and CQ) for reusable duodenoscope	Single HLD for reusable duodenoscope
Das et al, 2022 15	USA	Simulated cohort, hospital and published studies	To estimate cost-effectiveness of EXALT single-use duodenoscope versus current duodenoscope	Simulated cohort undergoing ERCP. Sub analysis for ERCP for Medicare patients in both hospital outpatient for TPT; hospital inpatient for NTAP. Age: 50 (20–90)	Single-use duodenoscope: (EXALT Model D, Boston Scientific Corp)	Reusable duodenoscope with current reprocessing methods (Standard HLD, CQ, EtO sterilization)
Kwakman et al, 2023 16	The Netherlands, USA	Medical center and published studies	To investigate costs associated with two scenarios in which single-use duodenoscopes are used in patients carrying MDROs	Two crossover scenarios, selected patients were treated with single-use duodenoscopes (ERCP) instead of regular reusable duodenoscopes, depending on MDRO carrier status. It was in the Erasmus Medical Centre: Rotterdam, US healthcare	Single-use duodenoscope	Reusable duodenoscope: (Pentax Medical, Dodewaard; Olympus, Zoeterwoude)
Nicolas-Perez et al, 2024 17	Spain	Hypothetical cohort hospitalized and published studies	To investigate cost-effectiveness of reusable duodenoscope versus a mixed option that also includes use of a single-use duodenoscope	A hypothetical cohort of 300 patients undergoing ERCP in Hospital Universitario de Canarias	Single-use duodenoscope: (EXALT Model D, Boston Scientific Corp: base-case; Ambu aScope Duodenum)	Reusable duodenoscope
Travis et al, 2020 18	Denmark	Clinical data and published studies	To investigate expected incremental costs and patient outcomes of using a reusable duodenoscope versus single-use Ambu aScope Duodenoscope	Patients undergone an ERCP	Single-use duodenoscope: (Ambu aScope Duodeno)	Reusable duodenoscope
CBDE, common bile duct exploration; CQ, culture and quarantine; CRE, carbapenem-resistant Enterobacteriaceae; ERCP, endoscopic retrograde cholangiopancreatography; EtO, ethylene oxide; FD, fully disposable; HLD, high-level disinfection; LC, laparoscopic cholecystectomy; MDRO, multidrug-resistant microorganism; NTAP, new technology add-on payment; PD, partially disposable; SRT, surveillance and reprocessing technique; TPT, transitional pass-through payment.						

### Economic evaluation methods and results

Details of economic evaluation methods and results are presented in Table 2 , Table 3 , and Table 4 .

**Table:** **Table 2** Detailed account of the economic evaluation methods - Part 1.

Authors/ year	Type of economic evaluation	Model type	Study perspective	Time horizon	Price year/currency	Discount	Authors/ year
Almario et al, 2015 12	Full economic evaluation (CUA)	Decision tree model using TreeAge software	Health care (hospital and payer)	1 year	2015/US$	N/A	CRE sepsis had a case fatality rate of 43%. Pretest probability of CRE-infected endoscope is 1%. probability of CRE transmission after ERCP with CRE-infected endoscope is 31%. Assumption that 73% of patients with CRE develops clinical symptoms. Proportion of initial admissions complicated by cholangitis requiring PTC tube placement is estimated to be 23%. QALYs
Barakat et al, 2022 13	Full economic evaluation (CUA)	Monte Carlo analysis model in R (used a multistate trial framework)	Health care	Post-ERCP lifespan of 7 years	Not stated/US$	Not stated	MDRO transmission rate after ERCP performed with MDRO-infected duodenoscope: 30%; rate of clinical symptom development in MDRO-infected patients: 50%; rate of survival after MDRO infection: 70%. QALYs lost by ERCP-transmitted infection. Environmental outcome stated
Bomman et al, 2021 14	Partial economic evaluation (cost analysis)	N/A	Not stated. Assumed from perspective of medical center	1 year	Not stated/US$	N/A	N/A
Das et al, 2022 15	Full economic evaluation (CUA)	Decision tree and Markov models	Health care	Lifetime Post-ERCP	2020/US$	2% for both cost and benefits	Probability of transmission of infection after exposure to contaminated duodenoscope scope with high-concern organism 0.4; probability of clinical infection requiring hospitalization after transmission of high-concern organisms associated with a contaminated duodenoscope 0.5; probability of different types of clinical infections from high-concern organisms: Pneumonia 0.3, Sepsis 0.2, UTI 0.2. QALYs
Kwakman et al, 2023 16	Partial economic evaluation (Break-even cost analysis)	Decision tree models involving two crossover scenarios	From perspective of a large tertiary care academic center	Not stated	Not stated/Euro, US$	Not stated	In the analyses, they used three different DAI infection risks; a bare minimum risk of 0.01% and maximum risks of 1% and 1.5%
Nicolas-Perez et al, 2024 17	Full economic evaluation (CUA)	Decision tree model using TreeAge software	Spanish National Health Service	1 year	2023/Euro	N/A	Risk of infection (probabilities): Infection by MDROs 0.01, cholangitis 0.01. QALYs
Travis et al, 2020 18	Full economic evaluation (CUA)	Decision-analytic Markov model	Hospital	10 years	Not stated/US$	Not stated	MDRO infection risk was estimated based on previously published literature. The reusable duodenoscope-related infection risk 1.21%. QALYs
CRE, carbapenem-resistant Enterobacteriaceae; CUA, cost-utility analysis; DAI, duodenoscope-associated infection; ERCP, endoscopic retrograde cholangiopancreatography; MDRO, multidrug-resistant organism; QALY, quality-adjusted life year; PTC, percutaneous transhepatic cholangiography; UTI, urinary tract infection.							

**Table:** **Table 3** Detailed account of economic evaluation methods - Part 2.

Authors/year	Outcomes/resource use and costs	Largest cost drivers	Excluded costs	Methods for estimating/collecting
Almario et al, 2015 12	Direct healthcare costs with each ERCP reprocessing strategy (per procedure), FDA-recommended endoscope: $69, culture and hold: $400, EtO sterilization: $1044; Hospital admissions	Admissions (e.g., PTC and LC/CBDE with major adverse event: $33,350)	Litigation, repair, disposal	Hospital admissions from Healthcare Cost and Utilization Project database. Physician services and procedures
Barakat et al, 2022 13	Per use cost of each approach: single reprocessing: $130.92, double reprocessing: $188.32, culture and hold: $386.67, EtO sterilization: $643.68, PD duodenoscopes: $251.28, and FD duodenoscopes: $2991.30. Environmental cost stated	Cost of management of cholangitis was based on 2 days of intensive care unit level care and 1 day of care in a step-down unit ($375,000); FD ERCP: $2,991.30	Repair	Parameters based on their tertiary care academic medical center and sterile processing division. Estimated comprehensive per use cost for each approach values from manufacturers prices
Bomman et al, 2021 14	Reprocessing cost: Single HLD: $80.47; Double HLD: $117.58; EtO gas sterilization: $296.49; Scope culturing-monitoring: $208.28. Materials: $49.59 single HLD, $74.83 double HLD, $51.28 EtO gas sterilization, $64.53 scope culturing-monitoring. Staff labor: $30.88 single HLD, $42.75 double HLD, $41.08 EtO gas sterilization, $33.95 scope culturing-monitoring. Labor: $109.82 scope culturing-monitoring, $204.13 EtO gas sterilization	Labor: EtO gas sterilization $204.13, culturing/monitoring $109.82	Training, AERs, leak testers, purchase, repair, disposal	Labor costs were categorized into 3 classes (low, mid, high): gastrointestinal tech III salary/hour. Estimated cost of capital as scope inventory/frequency of scope use per unit time, reprocessing costs required on a per-cycle basis; labor & staffing needs
Das et al, 2022 15	Standard HLD: $962; Double HLD: $977; CQ: $1,279; EtO gas sterilization: $1,561; EXALT Model D: $3,000; EXALT Model D in HIPD setting with NTAP estimated to offset $1,715 of SUD cost: $1,285; EXALT Model D in HOPD setting with TPT payment offsetting 100% of SUD cost: $0. Per use average cost of reprocessing: standard HLD: $200, double HLD: $215, CQ: $480, EtO sterilization: $1,180. Maintenance is included in reprocessing costs	Sepsis: $50,038 Cholangitis: $40,000 Misc. infection: $38,494 UTI: $33,400 Pneumonia: $30,432 EXALT Model D: $3,000	Caregiving time, physician services, litigation, disposal	Hospital costs of management of choledocholithiasis, post-ERCP contaminated scope; and infection adverse events cost: 2018 weighted national estimated inpatient diagnoses; and published literature
Kwakman et al, 2023 16	Price per ERCP procedure (reusable duodenoscopes): €180. Reusable: purchase (€39,309 per duodenoscope, €100 per ERCP procedure), reprocessing (€47 per ERCP procedure), maintenance (€2,500 (per year) per duodenoscope; €22 per ERCP procedure), surveillance culturing (€ 110 (per surveillance moment) per duodenoscope, €11 per ERCP procedure). Details of reprocessing cost was not stated	Costs for a duodenoscope-associated infection (cholangitis) total €6,774 per infection	Staff, specific instruments, productivity losses, disposal	Costs of materials, staff based on estimates from their own department at the Erasmus Medical Centre.US: They performed same break-even analyses for a US scenario
Nicolas-Perez et al, 2024 17	Single-use per-procedure cost: €2,900. Reusable per procedure cost: €1,333. Reprocessing, maintenance costs for reusable included but values not stated. Environmental cost stated	Single-use purchase: €2,900, Reusable ICU bed: €2,651	Not stated	Single-use scope. Reusable scope: average cost of ERCP in hospital and literature
Travis et al, 2020 18	Single-use: total per-procedure cost: $1,999. Reusable: weighted per-procedure cost in Markov model: $1,017. Purchase per use: single use: $1,995, reusable: $722. Equipment reprocessing: AER, supplies (e.g., sponges, brushes, and detergents), labor; reprocessing: $103, Reusable: microbiological sampling, culturing: $110, repair/maintenance: $83, Single: cost for a Box: $4	Endoscope-related MDRO infection cost $47,181; Purchase per use: single use: $1,999	Loss of earning, disposal	Per-procedure cost of reusable, cost of treating infection derived from a US multicenter micro-costing study. Infection risks, utilities, other parameters obtained from literature. Cost of single-use: Ambu
AER, automated endoscope reprocessor; CBDE, common bile duct exploration; CQ, culture and quarantine; ERCP, endoscopic retrograde cholangiopancreatography; EtO, ethylene oxide; FD, fully disposable; HLD, high-level disinfection; ICU, Intensive Care Unit; LC, laparoscopic cholecystectomy; PD, partially disposable; UTI, urinary tract infection.				

**Table:** **Table 4** Detailed account of economic evaluation methods and results - Part 3.

Authors/year	Results (ICER, e.g., incremental costs and outcomes)	Sensitivity analyses	Funding source	Declared conflicts
Almario et al, 2015 12	ERCP with FDA most cost-effective strategy. Both ERCP with culture and hold ($4,228,170/QALY) and ERCP with EtO sterilization ($50,572,348/QALY) strategies had unacceptable incremental costs/QALY. LC with CBDE being more costly, marginally less effective	A number of one-way sensitivity analyses. In sensitivity analysis, ERCP with culture and hold became the most cost-effective approach when pretest probability of CRE exceeded 24%	Not stated	All authors do not have any relevant disclosures or conflicts of interest
Barakat et al, 2022 13	At infection transmission rates <1%, PD duodenoscopes most favorable from a CUA. FD duodenoscope minimizes potential for infection transmission and is more favorable from a CUA than use of reprocessable duodenoscopes after single or double HLD at all infection rates, EtO sterilization for infection rates >.32%, and culture and hold for infection rates >.56%	Alternate scenarios of variation in hospital volume, QALY value, post-ERCP lifespan, environmental cost shifted cost-utility profiles. A sensitivity analysis for cost of treatment. With it, PD duodenoscope remained approach most favorable from a CUA	Not stated	Banerjee disclosed financial relationships: Consultant for Boston Scientific and Olympus. Other authors disclosed no financial relationships
Bomman et al, 2021 14	Compared to single HLD, double HLD increased costs by 47% ($80 vs $118). Similarly, CQ and EtO sterilization increased costs by 160% and 270 % ($80 vs $208 and $296)	Not stated	Not stated	Kozarek receives research support from Boston Scientific, National Institutes of Health. Thaker is a consultant for, receives research support from Boston Scientific. Muthusamy is a consultant for, receives research support from Boston Scientific, Medtronic; a consultant for Medivators, Interpace Diagnostics; a stockholder in Capsovision; receives honoraria from Torax Medical. Ross a consultant for Boston Scientific
Das et al, 2022 15	EXALT provided highest cost-savings to the hospital versus alternative strategies by maintaining QALY gains while decreasing estimated net cost	Sensitivity analyses were performed by varying cost estimates and clinical probabilities	Stated "None"	MC is an employee and stockholder of Boston Scientific Corp, which manufacturers the EXALT Model D single-use duodenoscope
Kwakman et al, 2023 16	In Dutch situation, single-use duodenoscopes needed to be priced at a maximum of € 140 to € 250 to break-even	Sensitivity analysis of DAI costs with a 20% and 40% increase of the separate variables included in the Erasmus MC	Not stated	The authors declare that they have no conflict of interest
Nicolas-Perez et al, 2024 17	Considering cholangiopancreatographies with single-use and reusable duodenoscopes at a cost of €2900 and €1333 respectively, and a 10% rate of single-use duodenoscopes, the ICER is € 3,967,917/QALY: base-case	Deterministic and probabilistic sensitivity analyses. 2- and 3-way sensitivity analysis confirmed increasing values of reusable ERCP and decreasing values of SUD-ERCP favored SUD-ERCP	Not stated	The authors declare that they have no conflict of interest
Travis et al, 2020 18	An additional cost of $28,525 per QALY, using an estimated cost of $1,995 per single-use duodenoscope. Thus, single-use duodenoscopes are cost-effective at a WTP threshold of $50,000 per QALY	Deterministic, probabilistic sensitivity analyses. A one-way sensitivity analysis to check results after changing cost of infection treatment. Cost $80,000-$85,000 to make single-use dominant	No financial support	HST, RVR, SA, and SL are employed by Ambu A/S, Ballerup, Denmark. NBL declared no conflicts of interest
CBDE, common bile duct exploration; CRE, carbapenem-resistant Enterobacteriaceae; CUA, cost-utility analysis; CQ, culture and quarantine; ERCP, endoscopic retrograde cholangiopancreatography; EtO, ethylene oxide; FD, fully disposable; HLD, high-level disinfection; LC, laparoscopic cholecystectomy; PD, partially disposable; QALY, quality-adjusted life year; SUD, single-use duodenoscope.				

#### Type of economic evaluation

There were five cost utility analyses 12 13 15 17 18 and two cost analyses 14 16 .

#### Type of modelling approach

Six studies employed a decision-analytic model 12 13 15 16 17 18 . Almario et al. used a decision tree model to evaluate a cohort of patients with common bile duct (CBD) stones to measure cost-effectiveness of four strategies for carbapenem-resistant *Enterobacteriaceae* (CRE) risk management 12 . Barakat et al. developed a Monte Carlo analysis model to assess cost utilities of six approaches to minimizing infection transmission for all patients having ERCP 13 . Das et al. constructed a decision tree model for patients with CBD stones to project consequences of contaminated scopes, with a Markov model to simulate colonization’s, infections, and treatment of infection 15 . Kwakman et al. used decision tree models involving two crossover scenarios based on presence or absence of multidrug-resistant organism (MDRO) carriage 16 . Nicolas-Perez et al. used a decision tree model to compare two ERCP modalities in a hypothetical cohort of 300 patients (case mix not reported) undergoing ERCP 17 . Travis et al. used a decision-analytic Markov model to compare the total cost and utilities 18 .

#### Perspective, time horizon, discount rate, and price year/currency

Six studies were from a healthcare provider perspective 12 13 15 16 17 18 . Bomman et al. did not state their perspective but it appeared to be that of healthcare provider 14 . The time horizons ranged from 12 months 12 14 17 to lifetime 15 . Three studies justified their choice of horizon 12 13 17 . Kwakman et al. did not state their time horizon 16 . In three studies 12 14 17 , discounting was unnecessary as the time horizons were 1 year 10 . Three studies did not state a discount rate 13 16 18 . Das et al. 15 reported an annual discount rate of 2% for both costs and benefits. The US Second Panel on Cost-Effectiveness in Health and Medicine recommends using a discount rate of 3% 19 . Only three studies specified their price year 12 15 17 . We converted currencies to 2024 British Pound Sterling (GBP) using the Bank of England exchange rates database and inflation calculator 20 21 . When price years were not reported, we used 1 year before publication.

#### Outcomes consequences measures

The most common outcomes were QALYs (n = 4) 12 13 15 18 and infection risk (n = 5) 12 13 15 16 18 .

**QALYs**

Five studies reported QALYs. Almario et al. 12 used QALYs as main outcome measure based on health state utilities from the Tufts Cost-Effectiveness Analysis Registry. Utility estimates were 0.50 (0.40–0.60) for CRE-related sepsis; 0.80 (0.70–0.90) for post CRE sepsis survival; 0.89 (0.79–0.95), 0.88 (0.78–0.95), and 0.76 (0.66–0.86), respectively, for ERCP without, with minor, and with major AEs. Barakat et al. 13 simulated QALYs lost by infection but the numbers lost were not reported. Das et al. 15 derived utility values of post-ERCP health states from literature, and estimated QALYs based on a post ERCP lifetime horizon. Reported utility value for an individual in full health was 1 (0.9–1.0), utility value with long-term colonization with high-concern organisms was 0.9 (0.85–1.0), disutility value of hospitalization for clinical infection was 0.06 (-0.02 to -0.15), and disutility value of Intensive Care Unit (ICU) admission was 0.11 (-0.06 to -0.34). QALY estimates for different reprocessing methods including standard HLD, double HLD, culture and quarantine, EtO sterilization, and for single-use EXALT Model D were 21.8938, 21.8938, 21.8904, 21.9093, and 21.9265, respectively. In Nicolas-Perez et al. 17 , QALYs were estimated to determine effectiveness of each approach. Utility estimates were 0.76 for cholangitis and 0.9 for without adverse outcome. Travis et al. 18 obtained utility scores through literature of 0.881 for no infection, 0.275 during infection, 0.639 during the 12 months after infection, and 0 for death. Based on a 10-year time horizon, estimated QALYs for reusable and single-use duodenoscopes were 6.58 and 6.70, respectively.

**Risk of infection and adverse events**

Six studies reported probabilities of ERCP-related infections using reusable endoscopes. Almario et al. 12 assumed that probability of endoscopes being contaminated by CRE was 1% ( **Supplementary Table 3** ), and that the chance of transmitting CRE following an ERCP procedure using a contaminated endoscope was 31%, and 73% of those patients with CRE would develop clinical infections. This indicated an overall infection rate of 0.2%. The case fatality rate for CRE-induced sepsis was 43%. In addition to exogenous infections caused by contaminated duodenoscopes, Almario et al. 12 also incorporated a proportion (23%) of admissions with preexisting cholangitis into their model. Barakat et al. 13 assumed a transmission rate of MDRO after ERCP using a contaminated duodenoscope of 30%, with 50% of infected patients developing clinical symptoms; the post-infection survival rate was 70%, derived from infectious disease experts and literature reviews. Das et al. 15 used published information and assumed that the duodenoscope contamination rate of high-concern organism after standard HLD was 6%, and the probability of infection from contaminated duodenoscopes was 40%, giving an overall infection rate of 2.4%. 50% of the infected patients required hospitalization, 10% to ICU. Among infections, probabilities for pneumonia, sepsis, and urinary tract infections (UTIs) were 30%, 20%, and 20%, respectively. Long-term colonization with high-concern organisms could occur. Kwakman et al. 16 used three risk levels of duodenoscope-associated infections (DAI): a minimal risk of 0.01% from reviews of Dutch DAI outbreaks, and higher risks of 1% and 1.5% from US analyses. Nicolas-Perez et al. 17 reported probabilities of infection by MDROs 0.01 and cholangitis 0.01. Travis et al. 18 estimated MDRO infection risks to be 1.21% for reusable duodenoscopes ( **Supplementary Table 3** ) and the post-infection mortality rate to be 19%.

#### Outcomes/resource use and costs

Resource use and costs varied according to the study purpose, case mix of patients, time horizon and the intervention and comparator.

**Total/average, purchase, reprocessing, maintenance/repair costs per procedure**

All studies included reprocessing costs (mainly labor, and equipment/material expenses). Six included purchase costs 12 13 15 16 17 18 . Repair and infection costs were respectively included by four 15 16 17 18 and six studies 12 13 15 16 17 18 . All studies stated that cost per procedure was greater using single-use endoscopes. Reprocessing cost was lowest using standard HLD and was progressively more expensive in the order of using double HLD, culture and hold, and EtO sterilization ( **Supplementary Table 4** ).

**Costs of ERCP-related infections**

Using the Healthcare Cost and Utilization Project database, Almario et al. 12 estimated cost of hospital admission for CRE to be $32,915 (£28,215) ($28,000 (£24,002) -$38,000 (£32,574)). Barakat et al. 13 , with a cohort of 800, reported a mean cost of $3125 (£2723) of cholangitis management for MDRO-infected patients who developed clinical symptoms based on 2 days of care in an ICU followed by 1 day of care in a step-down unit at their center. In their cohort of 10,000 patients, Das et al. 15 used the Premier Research database to derive treatment costs of high-concern organisms related infections of $50,038 (£49,404) for sepsis; $40,000 (£39,493) for cholangitis; $38,494 (£38,006) for infections in soft tissue, skin, and surgical site; $33,400 (£32,977) for UTI; and $30,432 (£30,046) for pneumonia. Kwakman et al. 16 calculated total treatment cost for a DAI of €6,774 (£6,452) at Erasmus Medical Centre in 2015. They also analyzed a US scenario in which DAI treatment cost was $20,119 (£18,435) based on costs from the US HCUPnet database. Nicolas-Perez et al. 17 obtained infection-related costs (hospital bed, ICU bed) from their hospital and length of hospital stay based on assumptions and published literature; however, total estimated cost for specific infection is not clear from the given data. Travis et al. 18 also used the HCUPnet database and estimated a mean cost of MDRO infection treatment of $47,181 (£46,242).

**Environmental costs**

Environmental impact was considered in only two studies. Barakat et al. 13 estimated environmental costs of ($35 (£31) for single reprocessing, $55 (£48) for double reprocessing, $100 (£87) for EtO sterilization, $60 (£52) for culture and hold, $80 (£70) for disposable endcap, and $150 (£131) for disposable duodenoscope) including chemicals, fumes/pollutants, and disposable solid waste. Nicolas-Perez et al. 17 estimated recycling cost of single-use and reusable duodenoscopes as €150 (£130) and €35 (£30) respectively.

**Largest cost drivers and excluded costs**

The largest cost drivers were managing infection for reusable scopes 12 13 15 16 17 18 and the purchase price for disposable scopes 13 15 17 18 . Some costs were excluded, most commonly disposal (n = 5) 12 14 15 16 18 , repair (n = 3) 12 13 14 , and purchase (n = 1) 14 costs. Costs due to litigation were not mentioned by any studies. Other costs excluded by some studies were: training, automated endoscope reprocessors (AERs), and leak testers 14 ; caregiving time and physician services 15 ; staff, productivity losses 16 ; and loss of earning 18 .

#### Sensitivity analysis undertaken

Bomman et al. did not perform any SA 14 . The six model-based studies conducted at least one SA. Almario et al. had one-way SA 12 . Barakat et al. used five SAs to explore the impact of alternative input values 13 . Das et al. used a probabilistic SA 15 . Kwakman et al. used two SA of alternative input values 16 . Nicolas-Perez et al. 17 and Travis et al. 18 had a number of deterministic/probabilistic SAs.

#### Synthesis of incremental costs, outcomes and cost-effectiveness evidence

Almario et al. 12 stated that ERCP with the Us Food and Drug Administration-recommended reprocessing was the most cost-effective strategy for mitigating CRE transmission risk. Barakat et al. 13 concluded that at infection rates < 1%, partially disposable (PD) duodenoscopes were most favorable. They noted that the fully disposable (FD) duodenoscope minimizes potential for infection transmission and is more favorable from a CUA than use of reusable duodenoscopes after single or double HLD at all infection rates, EtO sterilization for infection rates > 0.32%, and culture and hold for infection rates > 0.56%. Alternate scenarios of variation in hospital volume, QALY value, and post-ERCP lifespan shifted cost-utility profiles. Incorporating environmental cost reduced cost utility of FD duodenoscopes, but did not change the overall ranking of these approaches. Bomman et al. 14 indicated that compared with single HLD, adoption of double HLD increased costs of ERCP by 47%. Similarly, culture and hold and EtO sterilization increased costs by 160% and 270%, respectively. Das et al. 15 noted that device-specific reimbursement for EXALT (single-use duodenoscope) in the United States provided the greatest cost-savings to the hospital by decreasing estimated net costs of ERCP. Kwakman et al. 16 concluded that in the Netherlands, single-use duodenoscopes needed to be priced at a maximum of €140 (£133) to €250 (£238) to break even. Travis et al. 18 indicated an additional cost of $28,525 (£27,957) per QALY, using a cost of $1,999 (£1,959) per ERCP procedure using single-use duodenoscopes. They stated that single-use duodenoscopes are cost-effective at a willingness-to-pay (WTP) threshold of $50,000 (£49,005) per QALY. Nicolas-Perez et al. 17 in their base-case analysis stated that considering ERCP with single-use and reusable duodenoscopes at a cost of €2,900 (£2,518) and €1,333 (£1,157) respectively, and a 10% rate of single-use duodenoscopes, the ICER is €3,967,917 (£3,444,930)/QALY. Due to diversity of studies, meta-analysis was not feasible.

#### Generalizability

Das et al. 15 and Nicolas-Perez et al. 17 did not report on generalizability of their results. Almario et al. 12 noted that their findings from CBD stone patients may not be generalizable to all indications for ERCP, although they tried to generate results relevant to most settings. Barakat et al. 13 noted limitations of their model including cost and infection rate assumptions and potential lack of generalizability to lower-volume centers. Bomman et al. 14 did not include service costs, training costs, capital costs of reprocessing equipment, and other costs of maintaining the instruments and costs of enhanced surveillance and reprocessing techniques; therefore, their estimates are probably conservative compared with real life. They attributed the differences between their results and Barakat et al. 13 to variability of technique, labor, and materials costs and labor costs. Kwakman et al. 16 noted that costs for using reusable duodenoscopes differ greatly among institutions depending on ERCP volume, contracts with manufacturers, organization of reprocessing, MDRO prevalence rates, and time needed for testing MDRO carriage. Therefore, generalizability of their findings to other settings is uncertain. Travis et al. 18 noted that per-procedure cost of reusable ERCPs is highly dependent on annual volume, amount of capital equipment, and reprocessing setup.

#### Funding source and conflict of interest

Five studies did not state funding source 12 13 14 16 17 . Despite not declaring any financial support by two studies 15 18 , at the time of publication, several named authors were employed by the manufacturers of single-use endoscopes. Four studies reported potential conflict of interest 13 14 15 18 and three studies reported none 12 16 17 .

### Quality assessment of included studies

We used the 19-item CHEC checklist 11 ( Table 5 ). None of the studies fulfilled all the quality criteria although none were ranked as “worthless”. Most studies had very good reporting quality. The least well-addressed criteria were items 7 (all important/relevant costs identified?), 14 (discounted?), 18 (conflicts of interest?) and 19 (ethical and distributional issues?).

**Table:** **Table 5** Answers to the Consensus on Health Economic Criteria (CHEC) checklist 11 .

**Study**	**Item**																		
	**1**	**2**	**3**	**4**	**5**	**6**	**7**	**8**	**9**	**10**	**11**	**12**	**13**	**14**	**15**	**16**	**17**	**18**	**19**
Almario et al, 2015 12	Y	Y	Y	Y	Y	Y	N	Y	Y	Y	Y	Y	Y	NA	Y	Y	Y	Y	N
Barakat et al, 2022 13	Y	Y	Y	Y	Y	Y	Y	Y	Y	Y	Y	NC	NC	N	Y	Y	Y	N	N
Bomman et al, 2021 14	Y	Y	Y	Y	Y	NC	N	Y	Y	NA	NA	NA	NA	NA	N	Y	Y	N	N
Das et al, 2022 15	Y	Y	Y	Y	Y	Y	N	Y	Y	Y	Y	Y	Y	Y	Y	Y	N	N	Y
Kwakman et al, 2023 16	Y	Y	Y	Y	NC	Y	N	Y	Y	NA	NA	NA	NA	N	Y	Y	Y	Y	Y
Nicolas-Perez et al, 2024 17	Y	Y	Y	Y	Y	Y	N	NC	NC	Y	Y	Y	Y	NA	Y	Y	N	Y	N
Travis et al, 2020	Y	Y	Y	Y	Y	Y	Y	Y	Y	Y	Y	Y	Y	N	Y	Y	Y	N	N
Item 1 Is the study population clearly described?																			
Item 2 Are competing alternatives clearly described?																			
Item 3 Is a well-defined research question posed in answerable form?																			
Item 4 Is the economic study design appropriate to the stated objective?																			
Item 5 Is the chosen time horizon appropriate to include relevant costs and consequences?																			
Item 6 Is the actual perspective chosen appropriate?																			
Item 7 Are all important and relevant costs for each alternative identified?																			
Item 8 Are all costs measured appropriately in physical units?																			
Item 9 Are costs valued appropriately?																			
Item 10 Are all important and relevant outcomes for each alternative identified?																			
Item 11 Are all outcomes measured appropriately?																			
Item 12 Are outcomes valued appropriately?																			
Item 13 Is an incremental analysis of costs and outcomes of alternatives performed?																			
Item 14 Are all future costs and outcomes discounted appropriately?																			
Item 15 Are all important variables, whose values are uncertain, appropriately subjected to sensitivity analysis?																			
Item 16 Do the conclusions follow from the data reported?																			
Item 17 Does the study discuss the generalizability of the results to other settings and patient/ client groups?																			
Item 18 Does the article indicate that there is no potential conflict of interest of study researcher(s) and funder(s)?																			
Item 19 Are ethical and distributional issues discussed appropriately?																			
Y, yes; N, no; NA, not applicable; NC, not clear.																			

## Discussion

Our systematic review set out to assess all economic evaluations comparing single-use and reusable gastrointestinal endoscopes adopting various reprocessing strategies, although all included studies focused on duodenoscopes, where cross-infection was a particular concern. Lack of economic evaluation for other types of single-use gastrointestinal endoscopes was an important finding in its own right, because lack of cost-effectiveness evidence could serve as a precaution and a barrier to their wider adoption. Among the duodenoscope studies, per-procedure cost was higher using single-use endoscopes but three CUAs indicated that single-use endoscopes were more cost-effective. However, we have considerable reservations about the underlying clinical estimates ( **Supplementary Table 3** ).

Disutility value of hospitalization for clinical infection and disutility value of ICU admission reported by Das et al. 15 seemed very conservative. Utility scores during infection reported by Travis et al. 18 seemed very low considering that some were UTIs.

Infections after ERCP are a mixture of exogenous (organism from outside the body, for example from a contaminated endoscope) and endogenous (organism already present but moved from one part of the gastrointestinal tract into another) so assumptions of no infections should not be made for single-use endoscopes. Barakat et al. 13 and Travis et al. 18 made an assumption of no infection following ERCP with a single-use duodenoscope. However, Hutfless et al 22 , reported a 3.5% infection rate following ERCP with single-use duodenoscopes. Because the CUAs assumed no infection risk with a single-use scope, this is likely to have introduced significant bias into their analysis.

There was significant variation in reported rates of scope contamination, ranging from 1%. 12 to 6% 15 18 . Das et al. 15 cited several studies reporting 6% contamination rate. However, on review of the cited references, the authors could not find support for this assumption. However, the authors did note an FDA report from 2020 23 reporting similar rates of scope contamination.

Almario et al. 12 assumed a transmission rate from a contaminated duodenoscope of 31% but the references they cited do not support that figure, and the cited references have a much higher ratio of infection (73%) to colonization than other studies ( **Supplementary Table 3** ). Barakat et al. 13 assumed a similar figure for transmission but that only half result in clinical infections with half only colonized. Das et al. 15 assumes 40% transmission but based on “expert opinion” and it appears that all 40% are infections not colonizations. Therefore, we have concerns about the variability of assumptions in and findings of the CUAs.

Infection costs had the most impact on cost-effectiveness calculations. In the Barakat et al study 13 , the cost of cholangitis management was $3,125 (£2,723). Treatment costs in other studies ranged from €6,774 16 (£6,452) to $50,038 15 (£49,404). Per-procedure cost of reusable duodenoscopes is dependent on infection rates, ERCP volume, number of endoscopes and associated equipment, and reprocessing costs 18 24 25 . High purchase costs can be partially offset by labor, capital, and equipment/material expenses associated with different reprocessing approaches. Enhanced reprocessing methods could reduce infection risk but also increase cost of ERCP 13 .

The broader impact of endoscopy on the environment and ultimately human health is receiving increasing attention 26 27 . Le et al. 26 performed a life-cycle assessment comparing single-use and reusable duodenoscopes. Carbon emissions during manufacturing, reprocessing, and disposal of the scopes were evaluated, and emissions associated with single-use scopes were 24 to 47 times higher than reusable ones. Moreover, 90% of materials in single-use duodenoscopes are plastics, processing and accumulation of which may cause negative environmental and health impacts 17 28 29 . Only two of the included papers 13 17 incorporated environmental impacts in their economic evaluations, both as costs, without providing detailed rationale or calculations. The environmental costs they identified (waste or recycling costs) did not account for the full life-cycle of endoscopes and reprocessing strategies, potentially leading to underestimation of the environmental impacts.

There are challenges in assigning monetary values to environmental impacts but efforts are being made to develop methodologies for this type of economic evaluation 30 . Hensher 31 suggested the environmental impact can be incorporated into health economic evaluation, preferably using a cost-based approach to monetarily represent AEs caused by one additional ton of CO _2_ equivalent emission. This approach has been used by de Preux and Rizmie 32 to internalize greenhouse gas emissions as a cost within a cost-effectiveness analysis of different modes of hemodialysis delivery in the UK NHS, using a non-traded price of carbon (nTPC) of £52 (£78) per ton, which would add 0.7% to 1.3% to total costs.

In the studies reviewed, endoscope choice was primarily driven by healthcare costs. However, there is growing interest in sustainability, which has raised valid questions about conflicting findings and necessary trade-offs between cost-effectiveness and environmental impact. As part of our broader SUMU Endo project, we are currently assessing environmental impacts, including conducting a life-cycle assessment to translate these impacts into monetary terms for integration into cost-effectiveness analyses. In our view, future decisions regarding endoscope use are likely to be influenced by both healthcare costs and environmental impacts among other patient, clinical, and service delivery considerations. Therefore, determining the appropriate weight to assign to each factor will be crucial—particularly when they point in different directions. We believe that, ultimately, the decision may come down to local policy.

### Strengths and limitations of this review

We used a comprehensive search strategy of a broad range of electronic databases and grey literature. We quality assessed economic evaluations. Limitations included the shortcomings of the included studies, which are partly due to the evidence base at their timepoints. This systematic review may be affected by publication bias because unfavorable economic analysis of novel devices is less likely to reach publication. Some studies are supported by the manufacturers of single-use devices, and they may be reluctant to published unfavorable results.

## Conclusions

All studies stated that cost per procedure was greater using single-use endoscopes but three CUAs argued that using single-use endoscopes was more cost-effective. We have reservations about these studies due to the lack of support for their clinical effectiveness assumptions and inputs. Importantly, costs and consequences of single-use duodenoscopes would change if environmental costs and impacts were included. To achieve sustainable healthcare/balance sustainability and cost-effectiveness, life-cycle assessments that capture environmental impacts are needed, and future economic evaluations should incorporate these broader considerations beyond health.

## Supplementary Material

Supplementary Material Zusatzmaterial

## References

[1] Peery AF Crockett SD Murphy CC Burden and cost of gastrointestinal, liver, and pancreatic diseases in the United States: update 2021 Gastroenterology 2022 162 621 644 10.1053/j.gastro.2021.10.017 34678215 PMC10756322

[2] Ravindran S Marshall S Healey C P196 The national census of UK endoscopy services 2021 Frontline Gastroenterol 2022 71 A136 A136 10.1136/flgastro-2022-102157 PMC9555135 36250173

[3] Pioche M Pohl H Neves JAC Environmental impact of single-use versus reusable gastroscopes Gut 2024 73 1816 1822 39122363 10.1136/gutjnl-2024-332293 PMC11503130

[4] England, NHS. Improvement, NHS. Delivering a ‘Net Zero’National Health Service London. 2020 10.1136/bmjopen-2023-072944

[5] Ross AS Bruno MJ Kozarek RA Novel single-use duodenoscope compared with 3 models of reusable duodenoscopes for ERCP: a randomized bench-model comparison Gastrointest Endosc 2020 91 396 403 31679738 10.1016/j.gie.2019.08.032

[6] Ramai D Smit E Kani HT Cannulation rates and technical performance evaluation of commericially available single-use duodenoscopes for endoscopic retrograde cholangiopancreatography: A systematic review and meta-analysis Dig Liver Dis 2024 56 123 129 10.1016/j.dld.2023.02.022 37003844

[7] Cinelli M Coles SR Sadik O A framework of criteria for the sustainability assessment of nanoproducts J Cleaner Production 2016 126 277 287

[8] Page MJ McKenzie JE Bossuyt PM The PRISMA 2020 statement: an updated guideline for reporting systematic reviews BMJ 2021 88 105906 10.1136/bmj.n71 PMC8008539 33781348

[9] Chandler J Cumpston M Li T Cochrane handbook for systematic reviews of interventions London Cochrane 2019

[10] Drummond MF Sculpher MJ Claxton K Methods for the economic evaluation of health care programmes Oxford Oxford university press 2015

[11] Evers S Goossens M De Vet H Criteria list for assessment of methodological quality of economic evaluations Consensus Health Econ Criteria 2005 21 240 245 15921065

[12] Almario CV May FP Shaheen NJ Cost-utility of competing strategies to prevent endoscopic transmission of carbapenem-resistant enterobacteriaceae Am J Gastroenterol 2015 110 1666 10.1038/ajg.2015.358 26526083 PMC4721926

[13] Barakat MT Ghosh S Banerjee SJGE Cost utility analysis of strategies for minimizing risk of duodenoscope-related infections Gastrointest Endosc 2022 95 929 938 e2 10.1016/j.gie.2022.01.002 35026281

[14] omman S Kozarek RA Thaker AM Economic burden of enhanced practices of duodenoscopes reprocessing and surveillance: balancing risk and cost containment Endosc Int Open 2021 9 E1404 E1412 34466366 10.1055/a-1515-2591 PMC8382507

[15] Das A Cangelosi MJ Muthusamy VRJT A cost-effectiveness analysis of Exalt model D single-use duodenoscope versus current duodenoscope reprocessing methods Tech Innov Gastrointest Endosc 2022 24 16 25

[16] Kwakman JA Poley MJ Vos MC Single-use duodenoscopes compared with reusable duodenoscopes in patients carrying multidrug-resistant microorganisms: a break-even cost analysis Endosc Int Open 2023 11 E571 E580 37304249 10.1055/a-2064-9721 PMC10256319

[17] Nicolás-Pérez D Gimeno-García AZ Romero-García RJ Cost-effectiveness analysis of single-use duodenoscope applied to endoscopic retrograde cholangiopancreatography Pancreas 2024 53 e357 e367 10.1097/MPA.0000000000002311 38518062

[18] Travis HS Russell RV Adamsen S Early cost-utility analysis comparing the sterile single-use Ambu aScope Duodeno to reusable duodenoscopes. SSRN Electronic Journal 2020 10.2139/ssrn.3732784

[19] Sanders GD Neumann PJ Basu A Recommendations for conduct, methodological practices, and reporting of cost-effectiveness analyses: second panel on cost-effectiveness in health and medicine JAMA 2016 316 1093 1103 10.1001/jama.2016.12195 27623463

[20] Bank of England Inflation calculator https://www.bankofengland.co.uk/monetary-policy/inflation/inflation-calculator

[21] Bank of England Exchange rates https://www.bankofengland.co.uk/statistics/exchange-rates

[22] Hutfless S Shiratori Y Chu D Risk factors for infections after endoscopic retrograde cholangiopancreatography (ERCP): a retrospective cohort analysis of US Medicare Fee-For-Service claims, 2015–2021 BMJ Open 2022 12 e065077 10.1136/bmjopen-2022-065077 PMC9472111 36691191

[23] U.S. Food & Drug Administration (FDA) 522 Postmarket Surveillance Studies Database (Sampling and Culturing Study, Duodenoscopes) https://www.accessdata.fda.gov/scripts/cdrh/cfdocs/cfPMA/pss.cfm?t_id=354&c_id=3726

[24] Travis HS Ehlers LH Thornton JJPOA The total cost of reuseable duodenoscopes-are single-use duodenoscopes the future of ERCP? PharmacoEconomics - Open 2020 5 02 10.37421/pe.2020.5.125

[25] Bang JY Sutton B Hawes R Concept of disposable duodenoscope: at what cost? Gut 2019 68 1915 1917 30772837 10.1136/gutjnl-2019-318227 PMC6839801

[26] Le NNT Hernandez LV Vakil N Environmental and health outcomes of single-use versus reusable duodenoscopes Gastrointest Endosc 2022 96 1002 1008 10.1016/j.gie.2022.06.014 35718068

[27] Dhar S Chowdhury MAF Impact of environmental accounting reporting practices on financial performance: evidence from banking sector of Bangladesh Int J Asian Business Information Management 2021 12 24 42

[28] Zhang Q He Y Cheng R Recent advances in toxicological research and potential health impact of microplastics and nanoplastics in vivo Environ Sci Pollut Res Int 2022 29 40415 40448 10.1007/s11356-022-19745-3 35347608

[29] Sangkham S Faikhaw O Munkong N A review on microplastics and nanoplastics in the environment: Their occurrence, exposure routes, toxic studies, and potential effects on human health Marine Pollut Bull 2022 181 113832 10.1016/j.marpolbul.2022.113832 35716489

[30] Frew EJ Aligning health economics methods to fit with the changing world of public health Appl Health Econ Health Policy 2017 15 287 289 28258395 10.1007/s40258-017-0319-9

[31] Hensher M Incorporating environmental impacts into the economic evaluation of health care systems: Perspectives from ecological economics Resources Conservation Recycling 2020 154 104623

[32] de Preux L Rizmie D Beyond financial efficiency to support environmental sustainability in economic evaluations Future Healthc J 2018 5 103 107 10.7861/futurehosp.5-2-103 31098543 PMC6502566

