# Supplementary material for: Single-use versus reusable endoscopes in gastroenterology: Systematic review of full and partial economic evaluations
Source: Endosc Int Open. 2025 Jul 29;13:a26451463. doi: 10.1055/a-2645-1463 (PMC12372419; doi:10.1055/a-2645-1463)
Supplement: Supplementary file 1 — Supplementary Material [file 10-1055-a-2645-1463_26456864.pdf]

Table 1 MEDLINE (Ovid).
Ovid MEDLINE(R) ALL <1946 to September 24, 2024>
Date searched: September 25, 2024

1 exp Endoscopes, Gastrointestinal/ or exp Endoscopy, Digestive System/ 131759
2 (colonoscop* or sigmoidoscop* or duodenoscop* or esophagoscop* or oesophagoscop* or gastroscop* or enteroscop* or digestive scope? or GI scope? or GI endoscop* or choledochoscop* or cholangioscop* or pancreatoscop* or capsule endoscop*).kf,tw. 66028
3 1 or 2 161077
4 exp Gastrointestinal Diseases/di, dg, su 425645
5 exp Gastrointestinal Tract/ 706661
6 (gastr* or esophag* or oesophag* or upper GI or lower GI or bowel* or colon or colonic or duoden* or intestin* or stomach or digestive tract or endoscopic retrograde cholangi* or ERCP or ileum or ileal or jejun* or enteric).kf,tw. 1710056
7 endoscop*.kf,tw. 268919
8 Endoscopes/ or Endoscopy/ 66136
9 4 or 5 or 6 2082503
10 7 or 8 286668
11 9 and 10 162898
12 3 or 11 [GI endoscopes] 247690
13 Equipment Reuse/ 3256
14 (reusable or reuse* or reprocessed or re usable or re use* or re processed or "multiple use" or "multi use").kf,tw. 41615
15 13 or 14 [MU / reusable] 43267
16 Disposable Equipment/ 5369
17 (disposable or "single use" or "one time use").kf,tw. 22057
18 16 or 17 [SU / disposable] 24303
19 Environment/ or Carbon Footprint/ 70222
20 Environmental Pollution/ or Air Pollution/ or Air Pollution, Indoor/ or exp "Environmental Restoration and Remediation"/ or exp Water Pollution/ or exp Environmental Pollutants/ 468275
21 exp Climate Change/ or exp Greenhouse Effect/ 40224
22 exp "Conservation of Natural Resources"/ 121286
23 (environment* adj3 (impact* or conservation or cost? or resource?)).kf,tw. 55587
24 (climate change or global warming or greenhouse effect or net zero or climate impact? or greenhouse gas*).kf,tw. 95595
25 (carbon adj3 (emission? or impact? or neutral* or footprint)).kf,tw. 17946
26 (co2 adj3 (emission? or impact? or neutral* or footprint)).kf,tw. 9990
27 pollut*.kf,tw. 235829
28 sustainab*.kf,ti. 44655
29 (landfill or (waste adj2 disposa*)).kf,tw. 13198
30 (life cycle assessment* or life cycle analys?s or "cradle to grave analys*" or circular economy).kf,tw. 10880
31 exp Waste Products/ or exp Waste Management/ 167098
32 (waste adj2 manage*).kf,tw. 10661
33 (green* adj2 (endoscopy or surgery or gastroenterology)).kf,tw. 176
34 recycl*.kf,tw. 75861

35 19 or 20 or 21 or 22 or 23 or 24 or 25 or 26 or 27 or 28 or 29 or 30 or 31 or 32 or 33 or 34 [environmental impact] 964930
36 12 and (15 or 18 or 35) [GI endoscopes + reusable or single use or environmental] 1015
37 exp animals/ not humans/ 5261402
38 36 not 37 993

The MEDLINE search strategy was peer reviewed by Stevie van der Mierden, Information Specialist, Netherlands Cancer Institute and Maria Tan, University of Alberta, using the PRESS Checklist: PRESS Peer Review of Electronic Search Strategies. Last updated June 29, 2022. https://www.cadth.ca/press-peer-review-electronic-search-strategies-0

Table 2 Excluded studies and reasons based on code/criterion.

Author	Title	A/Year	B/Format	C/Language	D/Publication status	E/Population	F/Intervention	G/Comparator	H/Outcomes	I/Study type	J/Environmental effects
Agrawal et al, 2021	Sustainability of Single-Use Endoscopes				Narrative review and commentary of interest						
Bang Et al, 2019	Concept of disposable duodenoscope: at what cost?				Endoscopy news, comment						
Billi et al, 2002	Disposable versus reusable biopsy forceps in gastrointestinal endoscopy: A prospective, randomized, cost-effectiveness evaluation		Conference abstract								
Bourguignon et al, 2003	Disposable versus reusable biopsy forceps in GI endoscopy: a cost-minimization analysis						Biopsy forceps				
Burdick et al, 2004	Endoscope reprocessing and repair costs				Narrative review and commentary of interest						
Chahine et al, 2021	ID: 3522406 The cost of reusable duodenoscopes at a high-volume academic centre - is it time to switch to single-use duodenoscopes for ERCP?		Conference abstract								
Deprez et al, 2000	Disposable versus reusable biopsy forceps: a prospective cost evaluation						Biopsy forceps				
Dhar et al, 2021	Reducing low risk of transmissible infection in duodenoscopes: At what cost to the planet?				Postscript (letter)						
Ellrichmann et al, 2022	Single-use products in endoscopy-from consumables to single-use scopes			German							
Forte et al, 2011	Comparative cost-efficiency of the EVOTECH endoscope cleaner and reprocessor versus manual cleaning plus automated endoscope reprocessing in a real-world Canadian hospital endoscopy setting						Broncho-scopes				
Funk et al, 2014	High-level endoscope disinfection processes in emerging economies: financial impact of manual process				Narrative review and commentary of interest for all endoscopes						

	versus automated endoscope reprocessing					
Gordon et al, 2000	Reusable versus disposable forceps: the dilemma of cost and safety			Editorial		
Hernandez et al, 2022	Micro-cost estimates of procedures in a community-based ambulatory endoscopy center: when do we switch to single-use endoscopes?	Conference abstract				
Hoffman et al, 2023	Single-use gastroscope usage and implications in a high procedure volume facility: a case-study	Conference abstract				
Hoffman et al, 2024	Costs involved in compliance with new endoscope reprocessing guidelines			Narrative review		
Hogan et al, 2009	Cost-minimization analysis of jumbo reusable forceps versus disposable forceps in a high-volume ambulatory endoscopy center				Biopsy forceps	
Larsen et al, 2020	The hidden cost of colonoscopy including cost of reprocessing and infection rate: the implications for disposable colonoscopes			Endoscopy news, comments		
Le et al, 2022	Environmental and health outcomes of single-use versus reusable duodenoscopes					Sole focus on environmental effects
Lee et al, 2022	Single-Use Duodenoscopes for ERCP: Rationale, Feasibility, Cost, and Environmental Impact			Narrative review and commentary of interest		
Lee et al, 2022	POSA298 Economic Burden from Reusable Scope Repairs: A Literature Review	Conference abstract				
Lejeune et al, 2001	Disposable versus reusable biopsy forceps. A prospective cost analysis in the gastrointestinal endoscopy unit of the Dijon University Hospital		French			
Lim et al, 2012	Performance and cost of disposable biopsy forceps in upper gastrointestinal endoscopy: comparison with reusable biopsy forceps				Biopsy forceps	

Ma et al, 2018	Implementation of a systematic culturing program to monitor the efficacy of endoscope reprocessing: outcomes and costs			A systematic culturing program to monitor the efficacy of endoscope reprocessing
Muscarella et al, 2001	Biopsy forceps: disposable or reusable?		Narrative review and commentary of interest	
Muthusamy et al, 2018	Economic burden of emergent practices of duodenoscopes reprocessing and surveillance: Balancing risk-and cost-containment	Conference abstract		
Ofstead et al, 2017	A glimpse at the true cost of reprocessing endoscopes: Results of a pilot project		Pilot study, News/guidelines	
Peter et al, 2021	Single-use duodenoscopes: where are we and where are we going?		Narrative review and commentary of interest	
Petersen et al, 2000	Advantages of disposable endoscopic accessories		Narrative review and commentary of interest	
Prat et al, 2004	Reliability, cost-effectiveness, and safety of reuse of ancillary devices for ERCP			Endoscopy accessories
Rizzo et al, 2000	A performance, safety and cost comparison of reusable and disposable endoscopic biopsy forceps: a prospective, randomized trial			Biopsy forceps
Sicuro et al, 2020	Robotic Colonoscopy: Comparative Analysis of Costs Compared to Painless Conventional Colonoscopy			Technologies other than the ones specified.
Thiveaud et al, 2023	Costs of purchase, maintenance, microbiological control, and reprocessing of a reusable duodenoscope		Narrative review	
Tomiato et al, 2011	Disposable versus reusable biopsy endoscopic forceps: A cost analysis	Conference abstract		
Trindade et al, 2021	Single-use duodenoscopes and duodenoscopes with disposable end caps		Narrative review and commentary of interest	

Yang et al, 2000	A cost and performance evaluation of disposable and reusable biopsy forceps in GI endoscopy			Biopsy forceps
Zagalsky et al, 2015	Reuse of medical devices labeled for single use: Accepting and overcoming a need as a cost-saving measure in an ERCP unit	Conference abstract		

Thieme

Table 3 Checking clinical assumptions in CUAs.

	Endogenous versus exogenous infections	Proportion of duodenoscopes contaminated	Infection rate from contaminated duodenoscopes: infections and colorizations	Overall verdict
Almario et al, 2015 [1]	Not addressed	Assumption of 1%. "No data to support this"<br><br>(FDA 2020 report2 of 6% with older scopes and 0.5% with removable endcaps)	Assumptions of 31% transmission rate and 73% clinical infections after transmission.<br>Sources used<br>Transmission: Assumption not supported by references cited – Epstein [3], Alrabaa [4], Smith [5]. Epstein [3] reported on colonization detected by screening with no clinical infections reported. The proportion colonized was 26.5%.<br>Alrabaa4 transmission rate is about 19%. Of 10 cases, 3 were colorizations found by recall for screening. 7 were case detected in hospital by "an active rectal swab surveillance program" and it is not clear whether any had clinical infections<br>Smith [5] had 3 infections and one colonization from 17 patients, a rate of 23.5%<br>Clinical infection after transmission: 73% based on Alrabaa [4] and Smith [5]. Alrabaa [4] does not give any details of clinical infections. Smith 3 infections and one colonization so 75%<br>Later good quality study by Kim [6] (not available to Almario [1]) 14.4% transmission, 7.7% infected, 6.7% colonized.	Some concerns but Almario [1] quite dated so less evidence available
Barakat et al, 2022 [7]	Assumption of no infections with single-use scopes, but there will be endogenous infections	5% Source: FDA 2020 report [2]. The 5% is for high concern organisms. Rate for all organism 6.8%	30% transmission and 50% clinical infection in those with transmission, based on "infection disease consultation/literature-based".<br>Sources used: no references cited.	Major concern: endogenous infections and infection assumptions
Das et al, 2022 [8]	Not applicable as study started with contaminated scopes	6%<br>Sources cited: This figure is not supported by	Transmission rate assumed to be 40% "based on expert opinion".	Some concerns about transmission

		the references cited in table 2. Almario [1]1 assumed 1%. Kim [6] started with contaminated scopes so does not provide contamination rate. Rauwers [9] reported contamination of either 15% or 22% depending on measure used. However, modelling probably used the 6% reported by FDA [2]2	Frequency of clinical infection requiring hospital admission after transmission 50%. References cited Almario1 (which gave no relevant data), Kim [6] (7.7% acutely infected), Rauwers [9] (no relevant data) and “estimate”. So, the figure used is presumably from “estimate” based on clinical opinions of two authors.	rate and clinical infection rate
Nicolas-Perez et al, 2024 [10]	Not clear. Overall rate given for single-use scopes but not for reusable	6.1% based on good data from authors’ center	Cited Rauwers [9] as source but that study started with duodenoscopes known to be contaminated so not appropriate for all reusable scopes	Some concerns
Travis et al, 2020 [11]	Assumes almost no infections (rate of 0.000001% based on “estimate”) with single used duodenoscopes, but there will be endogenous infections	The approach did not used data on contaminated duodenoscopes but used overall infection risk of 1.2% after ERCP with re-usable duodenoscopes based on a review of infection risk by Larsen [12] (including Travis [11]) published only as a conference abstract with no details of which 6 studies were included. All 1.2% assumed to be due to MDR organisms<br>A later review of 51 ERCP studies by Deb [13] reported an infection rate of 0.8% which is reasonably close to 1.2%		Major concern regarding endogenous infections

CUA, cost utility analysis; FDA, Food and Drug Administration.

Table 4 Summary of total/average, purchase, reprocessing, maintenance costs per procedure of the studies.

**ERCPs using duodenoscopes**<br>**Cost categories stated per procedure**	**Ranged from**	**To**	**Other values in between**
**Single-use**			
Purchase cost = total/average cost	$1,999 [11] (£1959.21)	$3000 [8] (£2961.96)	€2900 [10] (£2517.77), $2991.30 [7] (£2606.74)
**Multiple-use**			
Total/average cost using standard HLD	$69 [1] (£59.15)	€1333 [10] (£1157.31)	$130.92 [7] (£114.09), €180 [14] (£171.45), $962 [9] (£949.8), $1,017 [11] (£996.76)
Total/average cost using double HLD	$188.32 [7] (£164.11)	$977 [8] (£964.61)	-
Total/average cost using culture and hold	$386.67 [7] (£336.96)	$1279 [8] (£1262.78)	$400 [1] (£342.88)
Total/average cost using EtO sterilization	$643.68 [7] (£560.93)	$1561 [8] (£1541.21)	$1044 [1] (£894.91)
Purchase cost	€100 [14] (£95.25)	$722 [11]* (£707.63)	-
Reprocessing cost (standard HLD)	€47 [14]† (£44.77)	$200 [8] (£197.46)	$80.47 [15] (£79.45), $103 [11]‡ (£100.95)
Reprocessing cost (double HLD)	$117.58 [15] (£116.09)	$215 [8] (£212.27)	-
Reprocessing cost (culture and hold)	$208.28 [15] (£205.64)	$480 [8] (£473.91)	-
Reprocessing cost (EtO sterilization)	$296.49 [15] (£292.73)	$1,180 [8] (£1165.04)	-
Maintenance/Repair cost	€22 [14] (£20.95)	$83 [11] (£81.35)	-

ERCP, endoscopic retrograde cholangiopancreatography; EtO, ethylene oxide; HLD, high-level disinfection.
*Includes capital investment.
†Cost of surveillance culturing: €11 has been considered separately.
‡Based on micro-costing.
Currencies are converted to 2024 GBP using the Bank of England exchange rates database and inflation calculator [16,17].
Purchase cost values of multiple-use scopes were not stated by four of the studies. (They were included in the total cost)[1,7,8,10]
Reprocessing cost values were not stated by three of the studies (they were included in the total cost) [1,7,10]. For one of the studies [8], maintenance/repair of reprocessing equipment were included in the reprocessing costs.

References

1 *Almario CV, May FP, Shaheen NJ* et al. Cost-utility of competing strategies to prevent endoscopic transmission of carbapenem-resistant enterobacteriaceae. Am J Gastroenterol 2015; 110: 1666
2 U.S. Food & Drug Administration (FDA). 522 Postmarket Surveillance Studies Database (Sampling and Culturing Study, Duodenoscopes) 2020 https://www.accessdata.fda.gov/scripts/cdrh/cfdocs/cfPMA/pss.cfm?t_id=354&c_id=3726.
3 *Epstein L, Hunter JC, Arwady MA* et al. New Delhi metallo-β-lactamase–producing carbapenem-resistant *Escherichia coli* associated with exposure to duodenoscopes. JAMA 2014; 312: 1447-1455
4 *Alrabaa SF, Nguyen P, Sanderson R* et al. Early identification and control of carbapenemase-producing *Klebsiella pneumoniae*, originating from contaminated endoscopic equipment. Am J Infect Control 2013; 41: 562-564
5 *Smith ZL, Oh YS, Saeian K* et al. Transmission of carbapenem-resistant *Enterobacteriaceae* during ERCP: time to revisit the current reprocessing guidelines. Gastrointest Endosc 2015; 81: 1041-1045
6 *Kim S, Russell D, Mohamadnejad M* et al. Risk factors associated with the transmission of carbapenem-resistant *Enterobacteriaceae* via contaminated duodenoscopes. Gastrointest Endosc 2016; 83: 1121-1129
7 *Barakat MT, Ghosh S, Banerjee SJGE*. Cost utility analysis of strategies for minimizing risk of duodenoscope-related infections. Gastrointest Endosc 2022; 95: 929-38. e2.
8 *Das A, Cangelosi MJ, Muthusamy VRJT* et al. A cost-effectiveness analysis of Exalt model D single-use duodenoscope versus current duodenoscope reprocessing methods. Tech Innov Gastrointest Endosc 2022; 24: 16-25
9 *Rauwers AW, Voor AF, Buijs JG* et al. High prevalence rate of digestive tract bacteria in duodenoscopes: a nationwide study. Endoscopy 2018; 67: 1637-1645
10 *Nicolás-Pérez D, Gimeno-García AZ, Romero-García RJ* et al. Cost-effectiveness analysis of single-use duodenoscope applied to endoscopic retrograde cholangiopancreatography. Pancreas 2024; 53: e357-e67
11 *Travis HS, Russell RV, Adamsen S* et al. Early cost–utility analysis comparing the sterile single-use Ambu® aScope^{TM} Duodeno to reusable duodenoscopes. SSRN Electronic Journal 2020
12 *Larsen S, Travis H, Russell R* et al. PMD1 rate of duodenoscope contamination and duodenoscope-related patient infection associated with endoscopic retrograde cholangio-pancreatography (ERCP): a systematic review and meta-analysis. Med Dev Clin Outcomes 2020; 23: S576
13 *Deb A, Perisetti A, Goyal H* et al. Gastrointestinal endoscopy-associated infections: update on an emerging issue. Digest Dis Sci 2022; 67: 1718-1732
14 *Kwakman JA, Poley MJ, Vos MC* et al. Single-use duodenoscopes compared with reusable duodenoscopes in patients carrying multidrug-resistant microorganisms: a break-even cost analysis. Endosc Int Open 2023; 11: E571-E80
15 *Bomman S, Kozarek RA, Thaker AM* et al. Economic burden of enhanced practices of duodenoscopes reprocessing and surveillance: balancing risk and cost containment. Endosc Int Open 2021; 9: E1404-E1412

16 Inflation calculator | Bank of England https://www.bankofengland.co.uk/monetary-policy/inflation/inflation-calculator
17 Exchange rates | Bank of England https://www.bankofengland.co.uk/statistics/exchange-rates
